# Eigenspace-based beamformer using oblique signal subspace projection for ultrasound plane-wave imaging

**DOI:** 10.1186/s12938-016-0244-4

**Published:** 2016-11-24

**Authors:** Saeid Aliabadi, Yuanyuan Wang, Jinhua Yu, Jinxin Zhao, Wei Guo, Shun Zhang

**Affiliations:** 1 Department of Electronic Engineering, Fudan University, Shanghai, 200433 China; 2 Key Laboratory of Medical Imaging Computing and Computer Assisted Intervention (MICCAI) of Shanghai, Shanghai, 200433 China

**Keywords:** Eigenspace-based minimum variance, Beamforming, Ultrasound plane-wave imaging, Oblique projection matrix, Signal subspace

## Abstract

**Background:**

The Eigenspace-based beamformers, by orthogonal projection of signal subspace, can remove a large part of the noise, and provide better imaging contrast upon the minimum variance beamformer. However, wrong estimate of signal and noise component may bring dark-spot artifacts and distort the signal intensity. The signal component and noise and interference components are considered uncorrelated in conventional eigenspace-based beamforming methods. In ultrasound imaging, however, signal and noise are highly correlated. Therefore, the oblique projection instead of orthogonal projection should be taken into account in the denoising procedure of eigenspace-based beamforming algorithm.

**Methods:**

In this paper, we propose a novel eigenspace-based beamformer based on the oblique subspace projection that allows for consideration of the signal and noise correlation. Signal-to-interference-pulse-noise ratio and an eigen-decomposing scheme are investigated to propose a new signal and noise subspaces identification. To calculate the beamformer weights, the minimum variance weight vector is projected onto the signal subspace along the noise subspace via an oblique projection matrix.

**Results:**

We have assessed the performance of proposed beamformer by using both simulated software and real data from Verasonics system. The results have exhibited the improved imaging qualities of the proposed beamformer in terms of imaging resolution, speckle preservation, imaging contrast, and dynamic range.

**Conclusions:**

Results have shown that, in ultrasound imaging, oblique projection is more sensible and effective than orthogonal subspace projection. Better signal and speckle preservation could be obtained by oblique projection compare to orthogonal projection. Also shadowing artifacts around the hyperechoic targets have been eliminated. Implementation the new subspace identification has enhanced the imaging resolution of the minimum variance beamformer due to the increasing the signal power in direction of arrival. Also it has offered better sidelobe suppression and a higher dynamic range.

## Introduction

Beamforming is a signal processing technique that enhances image quality by applying appropriate gains and delays to echo signals. The most implemented beamforming method in commercial and real-time ultrasound imaging systems is delay-and-sum (DAS). As a non-adaptive beamforming algorithm, DAS provides low imaging quality in contrast and resolution. To improve imaging quality, researchers have proposed adaptive beamformers that change the weights of the array elements or aperture using the characteristics of the received signals. The minimum variance (MV) and eigenspace-based methods are representative methods in adaptive beamformers.

Most adaptive beamformers are based on the linear constraint minimum variance (LCMV) method that was proposed by Capon in 1969 [1]. In LCMV, the weights are calculated based on the principles that the output signal-to-interference-pulse-noise ratio (SINR) is maximized, whereas the target signals remain undistorted. MV beamformer performance correlates to the signal-to-noise-ratio (SNR) of the signal. At high SNR, the MV beamformer offers significant improvements in image resolution. As SNR decreases, its performance approaches that of DAS [2]. Sasso and Cohen-Bacire [3] applied a spatial smoothing technique to decorrelate signal and noise. Synnevag et al. [4] proposed an MV beamformer that used the spatial averaging and diagonal loading techniques to increase the covariance matrix estimation accuracy. Izadi et al. [5] investigated the combination of coded excitation with the LCMV beamformer, and proposed a weighted Capon beamformer to increase the SNR without scarifying the contrast. Mohammadzadeh et al. [6] utilized the forward–backward minimum variance beamforming to enhance the covariance matrix estimation robustness without comprising imaging resolution. Kim et al. [7] used the principal component analysis (PCA) to reduce the computational complexity.

In recent years, eigenspace-based beamformers have been introduced into ultrasound imaging. This technique has been developed in earlier studies in radar imaging [8, 9]. Eigenspace-based beamformers use the signal subspace to modify the weight vector of the MV-based beamformer. The signal subspace is constructed by several selected eigenvectors of the input covariance matrix. The remaining eigenvectors correspond to the noise subspace. By eliminating the noise subspace, eigenspace-based beamformers obviously enhance imaging quality. Mohammadzade et al. applied this technique to medical ultrasound imaging [10]. They proposed the eigenspace-based minimum variance (EIBMV) beamformer, which significantly improved imaging contrast without scarifying the appreciable imaging resolution of the MV beamformer. The aperture weights of the EIBMV beamformer were found projecting the MV weight vector onto the estimated signal subspace. Mehdizadeh et al. [11] utilized the cross-spectral metric, (a metric of each eigenvector energy), to select the signal subspace dimension. To reduce the EIBMV calculation time, Zeng et al. [12] proposed a beamformer in the beam domain instead of the array domain.

Conventional eigenspace-based beamforming methods assumed that the signal component and interference and noise component are not correlated and used the orthogonal subspace projecting [10–15]. However, this assumption does not coincide with the reality that interference and noise are highly correlated with signal in medical ultrasound imaging. Scattering is the resource of generating both of information and noise. Also most of the noise consists of echoes of the transmitted pulse. Because of these reasons, the noise is actually highly correlated with the signal [15]. In other word, the no-correlation assumptions may not be valid in ultrasound imaging [16]. In this case, oblique projection is preferred to project the received echo onto a signal subspace along the noise direction that is oblique to the signal subspace. The oblique projection is regarded as proper method to extract desire signal while suppressing noise and interference. The oblique projection technique has been developed in earlier studies [17–19]. The performance of oblique projection in estimating the signal, suppressing the noise and interference and enhancing the SINR, greatly extended its application in array processing. [20, 21].

In this paper, we take oblique projection concept into the account of ultrasound imaging. To this end, a new beamformer that we call oblique eigenspace-based MV (OESMV) is proposed. The OESMV used oblique projection instead of orthogonal projection. To construct the oblique projection matrix, proper subspaces should be identified. To do so, we propose a new subspaces identification algorithm by investigating eigen-decomposition technique and signal-to-interference-pulse-noise ratio (SINR) concept. The weight vector will be estimated by projecting the LCMV weight vector onto the oblique projection matrix.

## Mathematic Background

### Signal and array model

In a uniformly spaced linear array of *M* elements, the received signal at the time instance *k* is given by

1 $$x\left( k \right) = s\left( k \right)\vec{a} + \vec{n}\left( k \right)$$

where *k* is the time instant, $x(k) = [x_{1} \left( k \right), \ldots , x_{M} \left( k \right)]^{\text{T}} \in C^{M\, \times \,1}$ is the array received signal, *s*(*k*) is the signal wave form, $\vec{a}$ is the steering vector, and $\vec{n}\left( k \right)$ is the sum of interference and the noise components. For each time instant (*k*), appropriate delays were applied to each channel to focus a certain interest point. In this way, the steering vector is defined as $a = [1, \,1, \ldots ,1]^{\text{T}} \in C^{M\, \times \,1}$, where (.)^T^ denotes the transpose; the beamformer output $y\left( k \right) \in C^{ 1 \times 1}$ is given by

2 $$y\left( k \right) = w^{\text{H}} (k)x_{d} (k) = \sum\limits_{j = 1}^{M} {w_{j}^{*} x_{j} \left( {k - \Delta_{j} (k)} \right)}$$

where *x*_*d*_ (*k*) is the time-delayed version of signals $x\left( k \right), \Delta_{j} (k)$; the appropriated time delay is applied to the channel *j* at time instant *k*. $w(k) = [w_{1} \left( k \right), \ldots , w_{M} \left( k \right)]^{\text{T}} \in C^{M\, \times \,1}$ is the weight vector.

### Minimum variance beamformer

Substituting (1) into (2), the output signal-to-interference-pulse-noise ratio (SINR) is given as [22],

3 $$SINR = \frac{{\sigma_{s}^{2} \left| {w^{\text{H}} a} \right|^{2} }}{{w^{\text{H}} R_{n} w}}$$

where $\sigma_{s}^{2}$ is the signal power, *R*_*n*_ is the noise and interference covariance matrix, w is the weight vector, and *a* is the steering vector. The optimum weight vector can be achieved by maximizing the SINR. It can be given by minimizing the output interference-pulse-noise power subject to the receiving signal without distortion.

4 $$\hbox{min} \;w^{\text{H}} R_{n} w,\quad {\text{subject to }}w^{\text{H}} a = 1.$$

Equation (4) can be solved by utilizing the Lagrange method. The optimal weight vector is computed as

5 $$w_{MV} = \frac{{R_{n}^{ - 1} a}}{{a^{\text{H}} R_{n}^{ - 1} a}}.$$

In practice R_n_ is not available. A common estimation for the covariance matrix is given by

6 $$R_{n} = x_{d} x_{d}^{\text{H}}$$

Spatial or temporal averaging is required to provide robust covariance matrix estimation. Spatial smoothing technique [3] is usually used to decorrelate the signal and noise. By applying the spatial smoothing technique, the covariance matrix is found by

7 $$\hat{R}\left( k \right) = \frac{1}{M - L + 1}\mathop \sum \limits_{p = 1}^{M - L + 1 } x_{d}^{p} \left( k \right)x_{d}^{p} \left( k \right)^{\text{H}}$$

where *L* is the subarray length, *M* − *L* + 1 is the number of overlapping subarrays, and $x_{d}^{p} (k) = \left[ {x_{d}^{p} \left( k \right),x_{d}^{p + 1} (k), \ldots , x_{d}^{p\, + \,L\, - \,1} \left( k \right)} \right]^{\text{T}}$ is the signal vector of the *p*th subarray. *L* is constrained to be smaller than *M/2*. Increasing *L* leads to signal and noise decorrelation; however, it may decrease signal intensity. The diagonal loading method is used to preserve the signal intensity and increase covariance matrix estimation robustness. In this regularization technique, a constant is added to the covariance matrix diagonal vector as $R = \hat{R} + \, \in {\text{I}}$, where I is the identity matrix and *R* is the diagonal loaded covariance matrix. ϵ is the diagonal loading factor, and its amount is usually set to be *μ* times the power in the received signals [2, 4],

8 $$\in \,=\, \mu \, *\,trace\;(\hat{R})$$

where $trace\;(\hat{R})$ is the sum of diagonal elements of the matrix $\hat{R}$, and *μ* is constant.

### Eigenspace-based minimum variance beamformers

By eigen decomposition technique, the input covariance matrix can be decomposed to the signal covariance matrix and noise and interference covariance matrix [9–14].

9 $$R = E\varLambda E^{\text{H}} = \mathop \sum \limits_{l = 1}^{L} \lambda_{l} e_{l} e_{l}^{\text{H}} = {\text{E}}_{\text{s}} \varLambda_{\text{s}} {\text{E}}_{\text{s}}^{\text{H}} + {\text{E}}_{\text{n}} \varLambda_{\text{n}} {\text{E}}_{\text{n}}^{\text{H}} = {\text{R}}_{\text{s}} + {\text{R}}_{\text{n}}$$

where Λ is the eigenvalue matrix, $\lambda_{1} \le \lambda_{2} \le \cdots \le \lambda_{L}$ are eigenvalues, and *e*_*1*_ is the *l*th eigenvector. The weight vector of the eigenspace-based MV methods is obtained by projecting the weight vector of the MV methods onto the signal subspace of the covariance matrix.

10 $$w_{ESBMV} = E_{s} E_{s}^{\text{H}} w_{MV} .$$

where $E_{s} E_{s}^{\text{H}}$, the signal subspace, contains the signal eigenvectors. In EIBMV [10], the signal subspace is constructed by the eigenvector contributions, which correspond to the large eigenvalues. The large eigenvalues are identified by the straightforward thresholding.

11 $$\lambda_{l} > \partial \lambda_{\hbox{max} }$$

where *λ*_*l*_ is the eigenvalue corresponding to eigenvector that is used to construct the signal subspace, *λ*_*max*_ is the maximum eigenvalue, and ∂ is a real positive multiplier. In practice, ∂ is chosen from 0.1 to 0.5. Another simple way to construct the signal subspace is to choose the eigenvectors whose related eigenvalues are *α* times the smallest eigenvalue. *α* is always chosen from 10 to 50.

12 $$\lambda_{l} > \alpha \lambda_{\hbox{min} }$$

In [11], the rank of signal subspace is chosen by identifying the largest *J* eigenvalues for which the sum of their cross-spectral metric is *β* times smaller than the total output signal power.

## Proposed Method

To further improve imaging contrast and resolution in eigenspace-based beamforming, we propose a novel beamforming method by employing the oblique projection. In this section, we modify an oblique projection matrix for ultrasound beamforming. To construct the oblique projection matrix, proper signal and noise subspaces must be known. To this end, a signal and noise subspace identification method is proposed. The weight vector of the OESMV is given by oblique projecting the MV weight vector onto the signal subspace along the noise subspace. Through the following subsections, subspace identification algorithm and oblique projection are described in details.

### Subspace identification

The large eigenvalues mainly correspond to the signal. However, there should be noise within the large eigenvalues. To identify the noise subspaces, we investigated the SINR concept. The eigenvectors of covariance matrix can be classified to three sets: the eigenvectors with largest eigenvalues and with high SINR set, the eigenvectors with largest eigenvalues and with low SINR set and the eigenvectors with small eigenvalues set.

In Eq. (3), the numerator represents the output signal power in DOA, and the dominator is the output interference-pulse-noise power. The weight vector, in numerator and dominator are not necessarily the same. Capon [1], considered the same signal and noise weight vectors. He calculated the weight vector, for which minimized the output interference-pulse-noise power subject to has the same direction of direction of arrival (DOA) (e.g. w^H^ a = 1). For first time, we consider signal subspace in numerator and noise subspace in dominator. The output SINR in (3), can be expressed as

13 $${\text{SINR}} = \frac{{{\text{P}}_{\text{y}}^{\text{s}} }}{{{\text{P}}_{\text{y}}^{\text{n}} }} = \frac{{\upsigma_{\text{s}}^{2} \left| {{\text{w}}_{\text{s}}^{\text{H}} {\text{a}}} \right|^{2} }}{{{\text{w}}_{\text{n}}^{\text{H}} {\text{R}}_{\text{n}} {\text{w}}_{\text{n}} }}$$

where ${\text{P}}_{\text{y}}^{\text{s}}$ is the output signal power, ${\text{P}}_{\text{y}}^{\text{n}}$ is the output noise power, w_s_ is the signal weight vector, and w_n_ is the noise weight vector. R_n_ can be estimated by Eqs. (8)–(9). To determine noise eigenvectors we calculate the SINR′ over largest eigenvalues. For the lth eigenvectors, the SINR′ is defined as

14 $${SINR^{\prime}}(\text{l}) = \frac{{\upsigma_{\text{s}}^{2} \left| {{w^{\prime}}_{\text{s}}^{\text{H}} \left( {\text{l}} \right){\text{a}}} \right|^{2} }}{{{w^{\prime}}_{n}^{\text{H}} \left( {\text{l}} \right){\text{R}}_{\text{n}} {w^{\prime}}_{\text{n}} \left( {\text{l}} \right)}},\;\;\;\;\left\{ { \left. {\text{l}} \right|\uplambda_{\text{l}} \ge \partial\uplambda_{ \hbox{max} } } \right\}$$

where $w^{\prime}_{s}$, the signal weight vector is obtained by projection the w_MV_ to lth large eigenvector.

15 $$w^{\prime}_{s} ({\text{l}}) = \left[ {{\text{e}}_{\text{l}} } \right]\left[ {{\text{e}}_{\text{l}} } \right]^{\text{T}} {\text{w}}_{\text{MV}} .$$

${w^{\prime}}_{\text{n}}$(l) is given by projecting the w_MV_ onto the lth noise subspace. The lth noise subspace is a union of the small eigenvectors and lth large eigenvector.

16 $${w^{\prime}}_{\text{n}} \left( {\text{l}} \right) = [{\text{e}}_{1} ,{\text{e}}_{2} , \ldots ,{\text{e}}_{{{\text{t}} - 1}} ,{\text{e}}_{\text{l}} ][{\text{e}}_{1} ,{\text{e}}_{2} , \ldots ,{\text{e}}_{{{\text{t}} - 1}} ,{\text{e}}_{\text{l}} ]^{\text{T}} {\text{w}}_{\text{MV}}$$

The noise and interference eigenvectors produce low energy in DOA. In other word, ${\text{e}}_{\text{n}}^{\text{T}} \text{a}$ and $\left| {{w^{\prime}}_{\text{n}} \text{a}} \right|^{ 2} \left| {{w^{\prime}}_{n}^{\text{H}} {\text{a}}} \right|^{ 2}$ suppose to be small values [12–16, 23]. On the other hand, noise eigenvectors increase the output interference-pulse-noise power $\left( {{w^{\prime}}_{n}^{\text{H}} \text{(l)}\,\text{R}_{\text{n}} {w^{\prime}}_{\text{n}} (\text{l})} \right)$. Consequently, the noise and interference eigenvectors provide a low SINR’.

To identify the signal subspace, we first select the largest eigenvalues by straightforward threshold according to (11). Then refined the selected eigenvectors by omitting the eigenvectors for which their output SINR’ estimated by (14), is η times smaller than maximum of SINR’:

17 $${SINR^{\prime}}({\text{l}}) < \upeta \, {\text{max}} \;({SINR^{\prime}}).$$

where max (SINR^’^) is the maximum value of SINR^’^ over all calculated SINR^’^ for each imaging point. η is constant between 0 and 1. Therefore the signal subspace, contains the eigenvector with large eigenvalue and with high SINR’;

18 $${\text{E}}_{\text{s}} = [{\text{e}}_{\text{t}} ,{\text{e}}_{{{\text{t}}\, + \,1,}} \ldots {\text{e}}_{{{\text{L}},}} ] \oplus [{\text{e}}_{{{\text{q}}1}} ,{\text{e}}_{{{\text{q}}2,}} \ldots ].$$

where [e_t_, e_t+1_,…, e_L_] are the eigenvectors with largest eigenvalues and [e_q1_, e_q2_,…]are the eigenvectors which satisfy (17). $\oplus$ denotes the exclusive or (XOR) operator. Remained eigenvectors are corresponded to the noise and interference. The noise eigenvectors correspond to two categories: the small eigenvalues and the eigenvectors with lowest SINR’. The noise basis vector can be given as

19 $${\text{E}}_{\text{n}} = [{\text{e}}_{1} ,{\text{e}}_{2,} \ldots ,{\text{e}}_{{{\text{t}} - 1}} ] \cup [{\text{e}}_{{{\text{q}}1}} ,{\text{e}}_{{{\text{q}}2,}} \ldots ].$$

where [e_1_, e_2_,…, e_t−1_] are the eigenvectors with smallest eigenvalues. ∪ denotes the union operator. It is easy to prove that E_s_ and E_n_ are orthogonal, then

20 $${\text{E}}_{\text{s}} {\text{E}}_{\text{s}}^{\text{T}} + {\text{E}}_{\text{n}} {\text{E}}_{\text{n}}^{\text{T}} = {\text{I }}$$

The subspaces of the oblique projection can be either orthogonal or non-orthogonal. To consider the signal and interference and noise correlation, we define a non-orthogonal subspace for oblique projection. In following we describe the identification method. Since the eigenvectors are linearly independent, any subspace of input covariance matrix can be spanned by its eigenvectors. Alternatively, any subspace may be identified as the set of some linear combination of eigenvectors. To respect the correlation between signal and noise, we define a non-orthogonal noise subspace by linearly combination of signal and noise eigenvectors. The non-orthogonal noise subspace is defined as

21 $$\widehat{\text{E}}_{\text{n}} = {\text{E}}_{\text{n}} {\text{J}}_{{{\text{k}},1}} - \uptau{\text{E}}_{\text{s}} {\text{J}}_{{{\text{m}},1}}$$

where J is all-ones matrix. τ is defined as correlation constant and its absolute ${\text{E}}_{\text{s}} \in {\text{C}}^{{{\text{n}}\, \times \,{\text{m}}}}$. The orthogonal signal subspace of ${\text{E}}_{\text{s}} \in {\text{C}}^{{{\text{n}}\, \times \,{\text{m}}}}$ and orthogonal noise basis matrix of ${\text{E}}_{\text{n}} \in {\text{C}}^{{{\text{n}}\, \times \,{\text{k}}}}$ are linearly independent and disjoint (m + k ≤ n). Consider the orthogonal projection matrix P_s_ = E_s_E_s_^T^, we have

22 $${\text{P}}_{\text{s}} {\text{E}}_{\text{s}} = {\text{E}}_{\text{s}}$$

23 $${\text{P}}_{\text{s}} \widehat{\text{E}}_{\text{n}} = {\text{P}}_{\text{s}} \left( {{\text{E}}_{\text{n}} {\text{J}}_{{{\text{k}},1}} - \uptau{\text{E}}_{\text{s}} {\text{J}}_{{{\text{m}},1}} } \right) = \uptau_{\text{s}} {\text{J}}_{{{\text{m}},1}} \ne 0$$

Therefore, $\widehat{\text{E}}_{\text{n}} \in {\text{C}}^{{{\text{n}}\, \times \,{\text{k}}}}$ and ${\text{E}}_{\text{s}} \in {\text{C}}^{{{\text{n}}\, \times \,{\text{k}}}}$E_s_ ∊ C^n × m^are linearly independent and disjoint but not orthogonal. We have used E_s_ and $\widehat{\text{E}}_{\text{n}}$ to construct the oblique projection matrix.

### Oblique projection of signal subspace

Consider two to full-rank complex matrices $\widehat{\text{E}}_{\text{s}} \in {\text{C}}^{{{\text{n}}\, \times \,{\text{m}}}}$ and ${\text{E}}_{\text{n}} \in {\text{C}}^{{{\text{n}}\, \times \,{\text{k}}}}$ as signal and noise basis matrices respectively; then the oblique projection operator Q_SN_ onto signal subspace along the noise subspace is defined as [18]:

24 $${\text{Q}}_{\text{SN}} = {\text{E}}_{\text{s}} ({\text{E}}_{\text{s}}^{\text{T}} {\text{Q}}_{{{\text{N}}_{\text{orthogonal}} }} {\text{E}}_{\text{s}} )^{ - 1} {\text{E}}_{\text{s}}^{\text{T}} {\text{Q}}_{{{\text{N}}_{\text{orthogonal}} }}$$

where ${\text{Q}}_{{{\text{N}}_{\text{orthogonal}} }}$ is the orthogonal complement projection matrix of the noise.

25 $${\text{Q}}_{{{\text{N}}_{\text{orthogonal}} }} = {\text{I}} - (\widehat{\text{E}}_{\text{n}}^{\text{T}} \widehat{\text{E}}_{\text{n}} )^{ - 1} \widehat{\text{E}}_{\text{n}}^{\text{T}}$$

where I is the identity matrix; where I is the identity matrix. The Q_SN_ has the following properties.

26 $$Q_{SN} = Q_{SN}^{2} \;\;\;\;\;\left( {\text{idempotent matrix}} \right)$$

27 $$Q_{SN}^{T} \ne Q_{SN}^{ - 1} \;\;\;\;\;\left( {\text{asymmetric matrix}} \right)$$

28 $$Q_{SN} E_{s} = E_{s}$$

29 $$Q_{SN} \widehat{E}_{n} = 0$$

Finally, the OESMV weight vector is obtained by projecting the MV weight vector onto the oblique projection matrix.

30 $${\text{w}}_{\text{OESMV}} = {\text{Q}}_{\text{SN}} {\text{w}}_{\text{MV}}$$

Both proposed eigenvector selection and oblique projection techniques, suppress the noise and interference component in direction of arrival. Thus by applying this algorithm, the OEBMV is able to provide a high output SINR.

### Implementation summary of algorithm

1. Here we give a brief summary of OESMV beamformer algorithm:
2. Calculate the time delay for each channel.
3. Compute the sample covariance matrix (R), using Eqs. (7)–(8).
4. Select the eigenvectors corresponding to the large eigenvalues, using Eq. (11).
5. Calculate SINR’ for each eigenvalues got by procedure 3, using Eq. (14).
6. Omit the eigenvectors with low SINR’ using Eq. (17). Compute E_s_, the orthogonal signal subspace using Eq. (18) and E_n_, the orthogonal noise subspace according to Eq. (19).
7. Estimate the non-orthogonal noise subspace $\left( {\widehat{\text{E}}_{\text{n}} } \right)$ with a proper choice of τ, the correlation constant, according to Eq. (21).
8. Construct Q_SN_ the oblique projection matrix, by employing E_s_ and $\widehat{\text{E}}_{\text{n}}$ got by procedures (3) and (7) respectively.
9. Compute the OESMV weight vector and beamformer output using Eq. (30) and (2).
10. The final beamformed image is obtained by repeating the above algorithm for each imaging point and can be illustrated by different dynamic ranges.

## Experiments and Results

We compared the performance of the OESMV with the DAS, LCMV, and EIBMV beamformers via simulation and real phantom studies. Since the one-steering-angle plane-wave imaging suffered from low SNR [23], we have evaluated the performance of proposed method in this worse case. For all experiments, spatial smoothing and diagonal loading techniques were implemented for adaptive beamformers. The DAS was implemented with rectangular apodization.

### Simulation study

Field II software [24, 25], was used to simulate the plane wave imaging for a linear array with 128 elements spaced at one-half wavelength (element width = 92.4 µm, height = 5 mm, kerf = 61.6 µm). The central and sampling frequencies were set to 5 and 100 MHZ, respectively.

#### Point target simulation

**Fig. 1:**
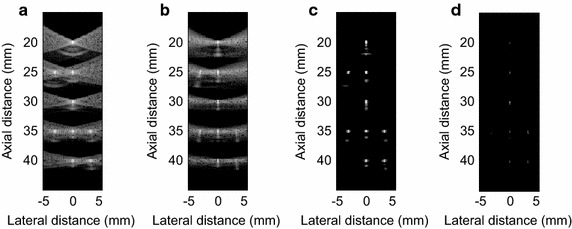
The beamformed response for simulated point targets, with 80 dB dynamic range. **a** The DAS, **b** the LCMV, **c** the EIBMV (∂ = 0.9) and **d** the OESMV (∂ = 0.9, η = 0.05, τ = 0.2)

Point target simulation was implemented to evaluate the imaging resolution and the side lobe suppression performance of the OESMV. In Fig. 1, nine points are located at the depth from 20 to 40 mm along the *x* = −3 mm, *x* = 0 mm, and *x* = +3 mm lateral direction.

To obtain the best resolution, a subarray size of L = 64 was used for spatial smoothing, and the loading factor *μ* was set to be (10 × L)−1. Figure 1 shows the resulting images over 80 dB dynamic range. In Fig. 1b, the LCMV offered a better resolution than the DAS in Fig. 1a. Both the LCMV and DAS beamformer provided low performance in side lobe suppression. As Fig. 1c illustrates, the EIBMV exhibited much better side lobe suppression than the DAS and LCMV beamformers. However, the EIBMV provided an imaging resolution similar to the LCMV with maximum threshold (∂ = 0.9). As shown in Fig. 1d, the OESMV offered a significant better lateral and axial resolution than that with both the LCMV and the EIBMV. This performance achieved due to eliminating the eigenvectors with largest eigenvalue and with the low SINR’ and using the projection. Also, applying the oblique projection, suppresses the artifacts alongside the axial direction of point targets. Applying the spatial smoothing technique creates this kind of artifact. The OESMV preserved the intensity of the hyperechoic targets. The normalized intensity of the target positioned at (x, z) = (0, 40) mm was −8.42 dB by the OESMV, −8.42 dB by EIBMV, and −8.49 dB by LCMV.

**Fig. 2:**
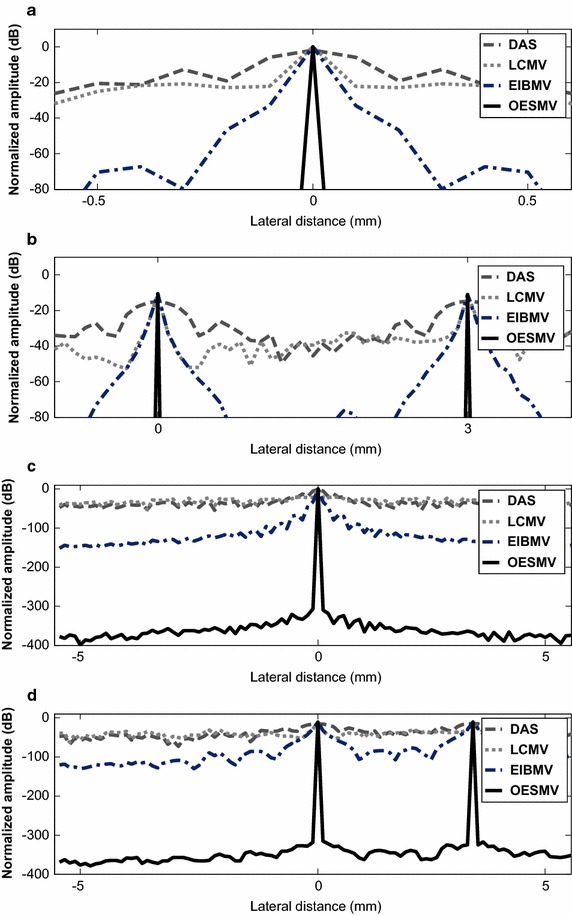
Lateral deviation of simulated point targets at the depth. **a** and **c** z = 20 mm, **b** and **d** z = 40 mm. **a**, **b** are shown with -80 dB and **c**, **d** with -400 dynamic ranges

Figure 2a, b show the lateral deviation of target points positioned at (x, z) = (0, 20) mm and (x, z) = (0, 40) mm with 80 dB dynamic range. The OESMV illustrated a beam pattern with significantly lower side lobes and narrower main lobe while preserving main lobe intensity. Figure 2c, d show the lateral profile of the beamformers at depth z = 20 mm and z = 40 mm with larger dynamic range. As can be seen the OESMV stretched the dynamic range significantly. Increasing the dynamic range is achievable for image visualization [26]. The performance of the imaging resolution and side lobe suppression were measured by the full-width at half-maximum (FWHM), peak-side-lobe (PSL) and floor level indexes [13]. The PSL is defined as the difference of the peak value of the first side lobe and main lobe. Table 1 gives the quantitative results. The FWHM of the OESMV was much better than that with the other beamformers (i.e. 10 times the EIBMV and the LCMV). The PSL results showed that the first side lobe level was obviously suppressed. The OESMV reached a much lower floor level compare with the EIBMV (i.e. −400 dB versus −151 dB). Based on the PSL and floor level results, OESMV provided a B-mode image with higher contrast and higher dynamic ranges than the EIBMV and the LCMV.

**Table 1:** Full-width at half-maximum (FWHM) and peak-side-lobe (PSL) results for beamformers

Beamformer	FWHM	FWHM	PSL	PSL
	(*x*, *z*) = (0, 20)	(*x*, z) = (0, 40)	(*x*, z) = (0, 20)	(*x*, z) = (0, 40)
*DAS*	0.2	0.5	−12.84	−19.34
*LCMV*	0.04	0.03	−20.93	−38.93
*EIBMV*	0.04	0.03	−67.3	−106.6
*OESMV*	0.003	0.003	−325.1	−331.5

*FWHM* full width at half maximum; *PSL* peak value of the first side lobe

#### Cyst simulation

A phantom cyst was simulated to evaluate the noise suppression, contrast enhancement, and dynamic range of the OESMV. The cyst had a 4 mm radius and was centered at (*x*, *z*) = (0, 35) mm. A subarray size of L = 48 was used in spatial smoothing, and the loading factor *μ* was set to be (10 × L) − 1. 350,000 scatterers have been generated by displacing randomly-distributed and zero-mean Gaussian amplitude.

**Fig. 3:**
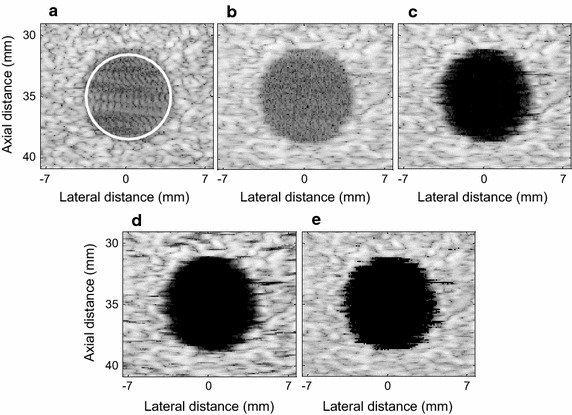
The beamformed response for the simulated cyst with a 80 dB dynamic range. **a** The DAS, **b** the LCMV, **c** the EIBMV (∂ = 0.03), **d** the EIBMV (∂ = 0.13) and **e** the OESMV (∂ = 0.03, η = 0.05, τ = 0.2)

Figure 3 shows the results with the 80 dB dynamic range. The DAS and LCMV beamformers illustrated poor contrast in Figs. 3a, 5b. As shown in Fig. 3c, by eigenvalue decomposition, the EIBMV removed a large part of the noise and the interference components. However, the cyst margin was still not very clear. As Fig. 3d illustrates, a larger threshold ∂ provided a clearer edge due to more noise suppression. However, this performance was achieved at the cost of creating more dark-spot artifacts in speckle region. As shown in Fig. 3e, the OESMV achieved sharper and better defined cyst boundaries compared with the image obtained by EIBMV. This significant clear cyst with sharp edges is obtained due to the removing eigenvectors with low SINR’ from signal subspace and the interference suppression by oblique projection. The oblique projection is able to preserve the desire signal intensity. As can be seen in Fig. 3e, OESMV provided a speckle pattern similar to that shown in Fig. 3c.

**Fig. 4:**
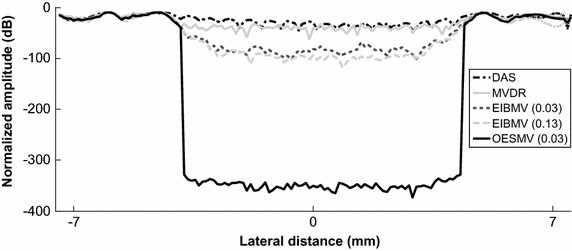
Lateral deviation of simulated cyst at the depth z = 35 mm

Figure 4 shows the variation of mean intensities of the OESMV, EIBMV, MV, and DAS beamformers on the lateral section at the axial axis center. The mean amplitude inside the cyst was significantly reduced by the OESMV due to more accurate noise and interference suppression, whereas the speckle intensity on both edges was better preserved than with EIBMV.

**Fig. 5:**
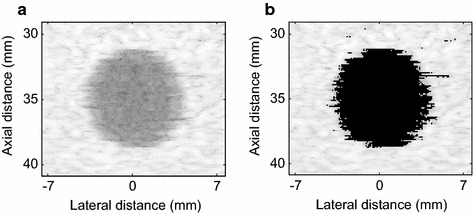
The beamformed response for the simulated cyst with a 200 dB dynamic range. **a** The EIBMV (∂ = 0.03), **b** the OESMV (∂ = 0.03, η = 0.05, τ = 0.2)

To show the dynamic range improvement by the OESMV, Fig. 5a, b illustrate the beamformed results of the EIBMV and the OESMV with a 200 dB dynamic range, respectively. As can be seen, the OESMV imaging quality was preserved for high dynamic range. This means that OESMV extended the dynamic range.

The contrast ratio (CR) and the contrast-to-noise ratio (CNR) were calculated as the quantitative measurement of the contrast. The speckle SNR was also calculated to assess the speckle statistics [14, 27],

31 $$CR = 20{ \log }\left( {\frac{{\varphi_{bck} }}{{\varphi_{cyst} }}} \right),$$

32 $$CNR = \frac{{\left| {\varphi_{cyct} - \varphi_{bck} } \right|}}{{\sqrt {\sigma_{cyst}^{2} + \sigma_{bck}^{2} } }},$$

33 $${\text{SNR}} = \frac{{\varphi_{bck} }}{{\sigma_{bck}^{1} }},$$

where ${{\varphi}}$_*cyst*_ and ${{\varphi}}$_*bck*_ are the mean amplitude (before log-compression) in the region of interest (ROI) of the cyst and speckle, respectively. In Fig. 3a, the ROI of the cyst and the speckle are indicated by a white circle. *σ*_*cyst*_^2^, *σ*_*bck*_^2^ are the relevant variances. Table 2 gives the CR, CNR, and SNR results for beamformers. Via eigen decomposition, the EIBMV provided a contrast enhancement of 34 and 27 dB over the DAS and the LCMV, respectively. The EIBMV with a larger threshold produced a higher CR of 10 dB, but a lower CNR and SNR. This is because it suppressed more noise inside the cyst, but shadowed the background. The OESMV eliminated the noise component inside the cyst better than EIBMV did, which led to larger CR. The larger SNR and CNR also showed that the OESMV beamformer could preserve the speckle pattern better than EISMV could, with the added benefit of reducing noise.

**Table 2:** CR, CNR and speckle SNR results of the simulated cyst

Beamformer	CR	CNR	SNR
*DAS*	−16.9228	1.3907	1.6377
*LCMV*	−24.2736	1.4591	1.5563
*EIBMV OESMV* (∂ = 0.03)	−50.8593	1.4110	1.4133
*EIBMV OESMV* (∂ = 0.13)	−60.9006	1.1616	1.1622
*OESMV* (∂ = 0.03, *η* = 0.05, τ = 0.2)	−77.8621	1.4432	1.4447

### Phantom study

Real data for the phantom imaging was carried out by employing the Verasonics ultrasound system (Verasonics Inc., Redmond, WA, USA) and multipurpose phantom (Model 040GSE CIRS, Inc., Norfolk, VA, USA), which contains hyperechoic targets and an anechoic cyst. The L11-4v (Philips Healthcare, Andover, MA, USA) linear probe, which consists of 128 elements with 0.3 mm pitch and 0.27 mm element width, was used for 1 acquisition plane wave imaging. Central and sample frequencies were set to be 6.25 and 26 MHz, respectively. The fractional bandwidth of the transducer was 60%, and the excitation pulse was a two-cycle sinusoid at the central frequency.

#### Point target phantom imaging

A wire targets with 0.1 mm diameters were positioned at the depths of *z* = 40 mm. To achieve the maximum resolution with a LCMV beamformer, a subarray size of *L* = 64 was used in spatial smoothing, and the loading factor *μ* was (10 × *L*)^−1^. Unlike simulation point target imaging, there was speckle in the target backgrounds.

**Fig. 6:**
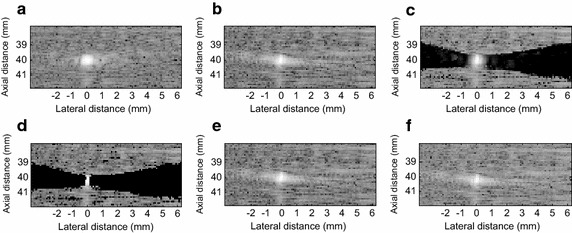
The beamformed response for the real wire phantom with 80 dB dynamic range. **a** The DAS, **b** the LCMV, **c** the EIBMV (∂ = 0.16), **d** the OESMV (∂ = 0.16, η = 0.05), **e** the EIBMV (∂ = 0.01), and **f** the OESMV (∂ = 0.01, η = 0.05, τ = 0.12)

The LCMV beamformer illustrated a higher resolution than the DAS in Fig. 6b. As can be seen in Fig. 6c, the EIBMV improved side lobe suppression by eliminating the contribution of small eigenvalues. The OESMV beamformer removed the low SINR eigenvectors and offered a better imaging resolution in Fig. 6d. The EIBMV and the OESMV both emphasized the points but also darkened the speckle and generated shadows surrounding the hyperechoic targets. These problems evolved due to straightforward thresholding with a large threshold (∂). To overcome this problem, we suggested setting a small threshold ∂ to mitigate the cancellation of the desired signal. The noise and interference suppression can then be derived by setting an appropriated SINR’ threshold (η) and a proper correlated constant (τ). In this way, OESMV improved the imaging resolution of MV-based beamformers by increasing the output SINR. As Fig. 6e shows, by setting a small ∂, the effectiveness of speckle shadowing was reduced but the EIBMV worked the same as the LCMV beamformer. In Fig. 6f, the OESMV represents a superior imaging resolution compared to LCMV and EIBMV without compromising speckle statistics.

**Fig. 7:**
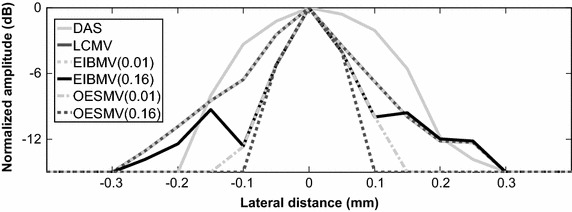
Lateral deviation of real point target at the depth z = 40 mm. The OSBMV, in both small and large straightforward thresholding has a narrower mainlobe at −6 dB narrower than that by the LCMV and the EIBMV

Figure 7 shows the lateral profile of beamformers at the hyperechoic target position. The DAS had the widest main lobe. By a large straightforward threshold (∂ = 0.16), the EIBMV and OESMV provided a narrower mainlobe than the LCMV. To preserve the speckle, ∂ should be set to a small value. In this way, the EIBMV with a small ∂ (∂ = 0.01) provided a beam pattern that fully matched the LCMV. But the OESMV offered a narrow main lobe even with a small ∂ (∂ = 0.01, η = 0.05, τ = 0.12). This means that the OESMV improved the resolution of the MV-based beamformer without comprising the speckle.

#### Cyst phantom imaging

**Fig. 8:**
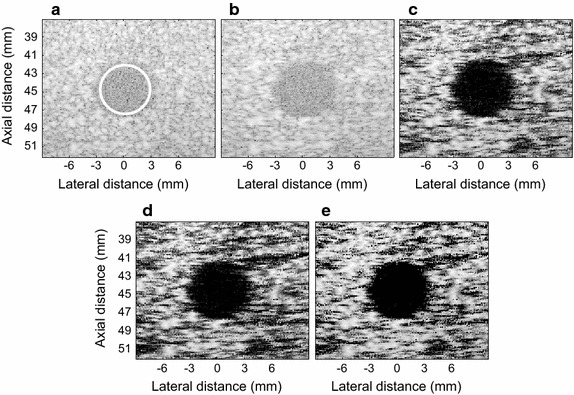
The beamformed response for the real cyst with 80 dB dynamic range. **a** The DAS, **b** the LCMV, **c** the EIBMV (∂ = 0.26), **d** the EIBMV (∂ = 0.3) and **e** the OESMV (∂ = 0.26, η = 0.05, τ = 0.11)

An anechoic cyst phantom with a 6.7 mm diameter was centered at (*x*, *z*) = (0, 45) mm. Figure 8 shows the beamformed images with 80 dB dynamic range.

The DAS and the LCMV beamformer suffered low image contrast. The EIBMV suppressed noise inside the cyst by signal subspace projection and enhance the contrast in Fig. 8c. Noise was further reduced inside the cyst, by increasing ∂ in Fig. 8d; however, the EIBMV darkened the speckle with a large ∂. The OESMV offered an image in Fig. 8e, with a clean cyst that had sharp boundaries, and a similar speckle pattern to the image in Fig. 8c.

For quantitative assessment, the CR, CNR and SNR were estimated. The ROI at the inside of the cyst was indicated by white circles. Referring to Table 3, the EIBMV increased the noise cancelling inside the cyst and improved the CR for 4 dB by increasing the threshold; however, the CNR and SNR were decreased. The OESMV provided a CR enhancement by 24 dB upon that with the EIBMV with small threshold (∂ = 0.26) and by 20 dB upon that with the EIBMV with large threshold (∂ = 0.37). Also, the OESMV provided a CNR and SNR enhancement by 0.01 and 0.01 dB respectively, upon that with the EIBMV with small threshold (∂ = 0.26) and by 0.13 and 0.13 dB respectively, upon that with the EIBMV with large threshold (∂ = 0.37). The higher CR, CNR and SNR results means that the OESMV suppressed noise inside the cyst better than EIBMV did, whereas preserving speckle statistics.

**Table 3:** CR, CNR and speckle SNR results of the real cyst

Beamformer	CR	CNR	SNR
*DAS*	−8.4068	0.9656	1.6102
*LCMV*	−8.7140	0.9431	1.5559
*EIBMV* (∂ = 0.26)	−50.8231	0.8022	0.8046
*EIBMV* (∂ = 0.37)	−54.0310	0.6803	0.6816
*OESMV* (∂ = 0.26, *η* = 0.05, τ = 0.11)	−74.5097	0.9119	0.9111

## Discussion

As an effective subspace method, oblique projection has been applied to medical ultrasound imaging successfully. We have proposed an eigenspace-based beamformer using oblique projection of signal subspace. Moreover, the eigen-decomposition and SNR analysis were investigated to provide a signal and noise subspaces identification algorithm. Real and simulation experiment results confirmed that the proposed beamformer displayed higher performance in imaging resolution, imaging contrast, speckle preservation, and dynamic range compared with the DAS, LCMV, and EIBMV beamformers. Point and wire target imaging in Figs. 1 and 6 showed the imaging resolution enhancement. The rejection of the low SINR’ components in the direction of the arrival provided higher output SINR, resulting in higher imaging resolution. Also, applying the oblique projection suppresses the noise and interference components significantly. As shown in Figs. 2 and 7, the OESMV provided a beam pattern with a narrower main lobe and significantly lower side lobes than that with LCMV and EIBMV. For anechoic cyst phantom imaging, as illustrated by Figs. 3, 5 and 8, the OESMV suppressed noise inside the cyst more than the EIBMV did, whereas restored signal intensity, resulting in a clearer cyst, and a better speckle pattern. Higher CR, CNR, and speckle SNR by the OESMV were reported upon the DAS, LCMV, and EIBMV beamformers.

Unlike the conventional eigenspace-based beamformers, the proposed subspace identification omits the largest eigenvalue from subspace, if it provides a low SINR’ in direction of arrival. This ability contributes to significant sidelobes suppression. The largest eigenvalue of those signals which originating from anechoic region or off-axis signal, provides a low SINR’. On contrary, the eigenvalue of those signals backcombing from hyperechoic targets, provides a high SINR’. Therefore the proposed subspace identification, is able mitigate the off-axis components while preserving the desire signal. On the other hand oblique projection technique, by projection the weight vector onto signal subspace along the direction which is parallel to noise and interference direction could provide better sidelobe suppression than that with orthogonal projection. As can be seen in Figs. 2 and 4, the OESMV overcompensates for the sidelobe effects in the anechoic regions, which significantly reduces the background level and gives the impression of empty space while preserved signal intensity.

To obtain an optimal image quality, the parameters (∂), (τ) and (η), should probably be adjusted for different scenarios. For example a low correlation constant (τ) and a small straightforward threshold (∂) recommended to preserve the signal intensity and speckle pattern. Increasing correlation constant (τ), provide a higher sidelobe suppression. However, this performance is achieved at the cost of signal distortion. For future work (τ) can be determined adaptively according to the received echo properties. By straightforward thresholding, both EIBMV and OESMV may bring dark spots on the speckle pattern. For large ∂, the OESMV may overestimate the noise at the cost of the speckle removal. To prevent speckle darkening, ∂ should be adjusted to a small value so that suppress noise inside the cyst. Afterward, by optimally adjusting the parameters (τ) and (η), the cyst edge will be distinguishable without scarifying the speckle. For too small ∂, even with a large (η), the OESMV could not eliminate the remaining noise. As similar problem, by straightforward thresholding, the Eigenspace-based beamformers suffered from shadowing alongside the hyperechoic targets and cysts. For this case the, to prevent the speckle darkening ∂ should be adjusted to a small value. Even with a small ∂, the OESMV is able to provide a high resolution performance by rejecting the low SNIR’ component in the direction of the arrival. As shown in Fig. 6f the OESMV could provide high-quality imaging resolution while preserving the speckle pattern surrounding the hyperechoic targets.

Eigenspace-based beamformers suffered from high computation complexity of O(L^3^) [12]. They have two major computational parts. One is the eigen decomposition and the other one is MV weight estimation. The OESMV added extra complexity to compute the SINR’ for each large eigenvalue. As an alternative way, the eigen-decomposition could be applied to DAS beamformer to decrease the calculation time. In this way, it is unnecessary to compute the inverse covariance matrix; the computation complexity will be reduced from O(L^3^) to O(L^2^). However, the DAS beamformer may degrade the imaging resolution quality.

## Conclusions

In conclusion, proposed signal and noise identification method and employing the oblique projection instead of orthogonal projection, have provided an accurate noise cancellation, which leads to increase plane wave Ultrasound image quality. According to the significant enhanced FWHM and PSL results, MV-beamformers imaging resolution was improved by increasing the SINR. The significant lowered side lobes, led to provide a plane wave B-Mode image with high dynamic range. The significant CR, CNR, and SNR results showed that OESMV improved the imaging contrast while preserving the speckle statistics. The OESMV beamformer also is able to solve the shadowing problem surrounding the hyperechoic targets.

## Abbreviations

DAS: delay and sum
MV: minimum variance
LCMV: linear constraint minimum variance
SNR: signal to noise ratio
EIBMV: eigenspace based minimum variance
OESMV: oblique eigenspace based minimum variance
SINR: signal to interference pulse noise ratio
DOA: direction of arrival
FWHM: full width at half maximum
PSL: peak side lobe
CR: contrast ratio
CNR: contrast to noise ratio
